# Struggling Thermal Stress Impacts on Growth Performance and Health Status of Newly Weaned Rabbits Using Nanoemulsion of *Origanum majorana* Considering the Economic Efficiency of Supplementation

**DOI:** 10.3390/ani13111772

**Published:** 2023-05-26

**Authors:** Ali Ali El-Raghi, Mahmoud A. E. Hassan, Nesrein M. Hashem, Sameh A. Abdelnour

**Affiliations:** 1 Department of Animal, Poultry, and Fish Production, Faculty of Agriculture, Damietta University, Damietta 34517, Egypt; 2 Animal Production Research Institute (APRI), Agriculture Research Center, Ministry of Agriculture, Dokki, Giza 12619, Egypt; 3 Department of Animal and Fish Production, Faculty of Agriculture (El-Shatby), Alexandria University, Alexandria 21545, Egypt; 4 Department of Animal Production, Faculty of Agriculture, Zagazig University, Zagazig 44519, Egypt

**Keywords:** rabbit, heat stress, inflammation, oxidative stress, immunity, economy

## Abstract

**Simple Summary:**

Climate change has strengthened the incidence of heat waves, causing significant losses in livestock units productivity and farm profit. Among different food producing animals, rabbits perform a crucial role in meat production in many countries worldwide. However, these animals are more sensitive to thermal stress compared with other livestock species, mainly due to the lack of functional sweet glands. Thus, maintaining rabbit farming systems under recent climate changes becomes a challenge. Phytochemicals may present an effective intervention to mitigate heat stress (HS) effects on rabbits via different modes of action. Recently, more attention has been paid for such substances, particularly, with the emergence of nanotechnology approaches. This technology has recently employed to improve the availability, solubility, and efficacy of phytochemicals, overcoming natural biological barriers and some industrial obstacles. In this sense, we explored the protective role of *Origanum majorana* (marjoram essential oil nanoemulsion, MEONE) against heat stress in growing rabbits, considering growth performance, immunity, and inflammatory and oxidative stress pathways. In addition, the economic efficiency of this nanotechnology-based intervention was estimated. The results indicate that the addition of 400 mg/kg diet of MEONE inhibited inflammatory biomarkers, DNA damages, and oxidative stress caused by heat stress, enhanced the growth performance, and relative economic efficiency of newly weaned rabbits.

**Abstract:**

With the recent trend of global warming, HS-instigated diminishing could extremely jeopardize animal health, productivity, and farm profit. Marjoram essential oil (MEOE) is a worthy source of wide range phytogenic compounds that may improve heat tolerance, redox and inflammatory homeostasis, and immunity of newly weaned rabbits, specifically if included in the diets in a nano form. One hundred newly weaned rabbits were randomly distributed into four homogeneous groups. The first group (control group) included rabbits that received basal diet without supplementation. In contrast, the other three groups included rabbits that received basal diets supplemented with 200 (MEONE200), 400 (MEONE400), and 800 (MEONE800) mg MEONE/kg diet, respectively. Among MEONE-treated groups and control groups, MEONE400 group showed the highest (*p* < 0.001) growth performance traits, including final body weight, average daily gain, feed efficiency, and the performance index. Compared to the control, all MEONE-supplemented groups possessed lower rectal temperatures and respiration rates, recording the lowest values in the MEONE400 group. The oxidative stress biomarkers and immunoglobulins G and M were significantly improved in the MEONE400 and MEONE800 compared with the control and MEONE200 groups. The addition of MEONE (400 or 800 mg/kg) decreased the concentrations of serum interleukin-4 (*p* = 0.0003), interferon gamma (*p* = 0.0004), and tumor necrosis factor-α (*p* < 0.0001) but significantly elevated (*p* < 0.001) the activity of nitric oxide, amyloid A and lysozyme. Liver functions (lower concentrations of liver enzymes) were significantly improved in all MEONE-treated groups compared to the control group. There was a considerable significant effect of dietary supplementation of MEONE400 on economic efficiency. In conclusion, the addition of 400 mg/kg to the diets of newly weaned rabbits can be recommended as an affective intervention to mitigate the negative impacts of HS.

## 1. Introduction

The whole temperature in the earth is predictable to be increased by 4 °C in the subsequent 100 years [1]. Increasing global temperatures will have deleterious consequences on the livestock sector by reducing the quality and quantity of feedstuff, augmented competition for natural resources, deprivation of biodiversity, the spread of livestock syndromes, and augmented heat stress (HS) [2].

Among these obstacles, HS seems the largest threats fronting animal populations in current times, and the deleterious effects of HS have begun to alter animal health, physiology, and behavior, ultimately with negative consequences for animal fecundity and survival [1]. Rabbits are categorized as the best meat producers due to their high meat quality, superior growth rates, and feed efficacy [3]. In addition, rabbit breeding is a veritable approach of decreasing poverty due to its low cost of breeding in developing countries. Moreover, the consumption of rabbit meat does not contravene any religious or social taboos. Global rabbit meat production is presently assessed at 1,482,441 tons equivalent carcasses [4].

In this scenario, there is an emergent concern in escalating the rabbit industry as an economical and environmentally sustainable livestock farming system [5]. With this regard, HS has substantial impacts on rabbit production and its physiological aspects, especially in tropical and subtropical areas. According to many reports, the thermoneutral zone of rabbits is 15–25 °C; while higher temperatures cause discomfort and/or stress conditions [6,7]. Ardiaca et al. [8] indicated that the body temperature of rabbits can increase up to 40 °C in stressful situations without being pathological. However, above 40 °C, cell membranes start to be destroyed by protein denaturation [9]. From 24 °C onwards, weaned rabbits, during the fattening period, start to have respiratory problems, with fatigue, increased heart rate, lack of appetite, and decreased basal metabolism. Thus, newly weaned rabbits are expected to be more sensitive to HS, as they usually suffer weaning psychological shock and severe physiological/nutritional changes post-weaning, making them more susceptible to negative impacts of HS [7]. Many reports have shown that HS can negatively affect economic traits, including growth, feed efficiency, and meat quality as well [10,11]. In addition, HS may interfere with growing rabbits welfare and health by impairing the immune system and redox status and evoking inflammatory reactions [12,13]. For this concept, it is important to bring our attentions toward establishing good management for growing stage in rabbits. Several approaches have been employed to diminish the detrimental effects of HS in growing rabbits’ improve health, welfare and meat quality [6,14,15], and maximize the rabbit industry economy.

*Origanum majorana* (marjoram) is one of the most prevalent condiments applied for many food preparations, agricultural, pharmaceutical, and biomedical uses, aiming its essential oils [16]. The marjoram essential oil nanoemulsion (MEOE) has strong antioxidant, anti-inflammatory, and antimicrobial abilities [17]. It has considerable amounts of terpinenes (e.g., terpinen-4-ol, trans-sabinene-hydrate, γ-terpinene, α-terpinene, carvacrol, and thymol) [18,19,20].

Marjoram essential oil alleviated the renal damages induced by ivermectin [21] via levitation antioxidant capabilities, lessening inflammation in rabbits. Moreover, *Origanum majorana* has beneficial effects on feed efficiency, antioxidant abilities, growth indices, and immunological reactions in lamb and fish [22,23,24,25].

Nevertheless, it is well known that the use of essential oils as a dietary supplementation is restricted due to their limited permeability, solubility, bioavailability, and storage instability. Recent studies have shown that transforming phytochemicals into nano form can confer them several biological and industrial advantages, making them more kinetics in the biological systems and more suitable for handling under industrial conditions [26,27].

Based on previous findings, we hypothesized that the dietary inclusion of MEONE may have protective effects against HS by boosting the growth and improving the health of newly weaned growing rabbits. Moreover, we considered the economic efficiency of the supplement to prove the cost efficiency of the supplementation when a technology, such as nanoemulsion, was used.

## 2. Materials and Methods

### 2.1. Preparation of Marjoram Essential Oil Nanoemulsion

The MEOE was purchased from the pure life company, Giza, Egypt. A single layer of MEONE, oil-in-water, was prepared [28]. In brief, nanoemulsions were prepared from MEOE 2.5 mL and surfactant (tween 80) mixture by slowly dropping water (up to 10 mL) appointed by a magnetic stirrer at 25 °C. The dropping rate of water was preserved persistent at around 1.0 mL/min. Then, the emulsion was scattered for 30 min through an ultrasonic bath (Sonix, Springfield, VA, USA, SS101H230). It was additionally homogenized by employing an ultrasonic probe (Serial No. 2013020605, Model CV 334) involved to a homogenizer (Sonics Vibra-cell™, Model VC 505, Inc., USA) under the subsequent conditions: amplitude: 60%, timer: 5 min, and pulser: 1 s ON/1 s OFF to yield nano emulsions.

### 2.2. Physichochemical Properties of MEONE

The internal morphology of the freshly prepared MEONE was visualized using transmission electronic microscope (TEM, JEOL JEM-2100, JEOL Ltd., Tokyo, Japan) at 160 kV. Digital Micrograph and Soft Imaging Viewer software (Gatan Microscopy Suite Software, version 2.11.1404.0) were utilized to complete the image capture analysis process. Nano emulsion average vesicular sizes (Z-average), polydispersity index (PDI) and surface charge of nanoemulsion particles (Z-potential) were measured using Zetasizer Nano ZS analyzer (Malvern Instruments, Malvern, UK).

### 2.3. Animals, Housing and Experimental Design

All the procedures that used the rabbits for this trial were approved by the scientific committee of the Institutional Animal Care and Use Committee (IACUC) of Zagazig University (Approval number: ZU-IACUC/2/F/367/2022).

One hundred weaned New Zealand White (NZW) male rabbits (707 ± 14.35 g, six weeks of age) were involved in this study. The weaned rabbits were acquired from the Rabbit Research Unit, Faculty of Agriculture, Zagazig University, Egypt. All rabbits were kept in the same environmental and management conditions. The animals were housed in galvanized wire battery cages (50 × 45 × 40 cm^3^) provided with conventional feeders and an automatic system of nipple drinkers in a well-ventilated rabbitry. The growing rabbits were randomly distributed into four groups, the control group, where rabbits received basal diet without supplementation, and three treated groups that included rabbits receiving basal diets supplemented with 200 (MEONE200), 400 (MEONE400), and 800 (MEONE800) mg MEONE/kg diet, respectively. The experimental period lasted eight weeks (July and August, presenting summer season). Growing rabbits were fed with basal diets formulated to congregate the nutrient requirements as endorsed by NRC [29]. The ingredients and chemical composition of the basal diet is presented in Table 1. The diet ingredients are free of antibiotics. During the experimental period, ambient temperature (AT) and relative humidity (RH) were assessed using a hygrothermograph (ST-50A, SEKONIC, Tokyo, Japan) placed in the animal facility. The hygrothermograph was placed above (30–50 cm) the cages for measuring the RH and AT at the constant time every day. These parameters (RH and AT) were recorded daily at 14.00 PM. Both variables were used to calculate the temperature humidity index (THI) values and thereby detect the severity of HS during the experimental period. The following question was used for detecting the THI values THI = db − [(0.31 − 0.31(RH)] × [(db °C − 14.4)], where db is the dry bulb temperature (AT) in Celsius and RH is the percentage relative humidity. The THI values were subsequently classified as follows: <27.8 = absence of heat stress; 27.8 to 28.9 = moderate heat stress; 28.9 to 30.0 = severe heat stress, and >30.0 = extremely severe heat stress [7]. The previous method was used to detect the severity of HS in rabbits.

### 2.4. Growth Performance and Physiological Responses

Live body weight (LBW) of individual rabbits was determined weekly, and average daily weight gain (DWG) was calculated according to the following equation: DWG = [(Initial body weight − Final body weight)/duration]. Feed intake (grams per rabbit) was weekly determined during the whole experimental period. Feed intake (FI) was determined by weighing the residuals of daily offered feed. The feed conversion ratio (FCR; g feed/g gain) and performance index (PI, % = [(FBW, Kg × 100)/FCR] were estimated accordingly.

Rectal temperature (RT; °C) and respiratory rate (RR; breath/min) were weekly measured. RT was assessed by inserting a digital thermometer (Model No.: YF-160A, TYPE-K) to a depth of approximately 4 cm into the rectum for 1 min. At the same time, RR was evaluated by visually calculating the number of movements of the flanks of the rabbits in a resting position for 1 min with a stopwatch. The thermometer was disinfected between each animal via apply rubbing alcohol (60%) with a cotton swab.

### 2.5. Slaughtering and Carcass Characteristics

At the end of the treatment, ten rabbits from each experimental group were randomly picked out and fasted for 12 h and immediately slaughtered after weighing them individually. We followed the Islamic method for slaughtering the rabbits (we used sharpness knife qualified person for slaughtering, then used a special words during the slaughtering), as indicated in a recent study by Bouzraa et al. [30]. The viscera, tail, and pelt were removed after complete bleeding, and then the carcass and its components were weighed as total edible parts. The edible giblets (heart, liver, and kidneys) were weighed as a percentage of live body weight. Similarly, the dressing percentage was calculated by dividing the hot-dressed carcass weight by pre-slaughter weight and expressed as a percentage.

### 2.6. Blood Sampling and Analyses

The blood samples were collected form six rabbits/group the marginal ear vein in sterilized blank tubes. The blood samples were left for 2 h, allowing clot formation, then the serum samples were obtained by centrifugation at 3000× *g* for 15 min (T32c; Janetzki, Wallhausen, Germany). Serum samples were detached and preserved at −20 °C for further analyses.

For assessing the redox status of rabbits, the activities of Total antioxidant capacity (TAC), superoxide dismutase (SOD), and glutathione (GSH) were determined using specialized quantitative sandwich ELISA kits (TAC: MBS8807700, SOD: MBS8807589, and GSH:MBS9718983, My BioSource, San Diego, CA, USA) following the guideline instructions. Moreover, the concentrations of malondialdehyde (MDA) and protein carbonyl (PC) were determined as lipid and protein oxidation biomarkers, respectively, using a specialized ELISA kit (MDA: MBS8806802, PC: MBS2601439, My BioSource, San Diego, CA, USA). At molecular level, the concentration of n 8-hydroxy-2′-deoxyguanosine (8-OHdG) as an indicator for oxidative DNA impairment was assessed using a commercial competitive sandwich ELISA kit (Trevigen, Gaithersburg, MD, USA).

For immune function evaluation, the concentrations of immunoglobulin M (IgM) and G (IgG) were assessed by ELISA kits [31], and the concentration of myeloperoxidase (MYO: MBS724170, My BioSource, San Diego, CA, USA) was also determined.

For inflammatory assessment and liver function, the levels of interferon gamma (IFNγ), tumor necrosis factor (TNF-α), and interleukin 4 (IL-4) in rabbit serum were determined using commercially available sandwich ELISA kits (IFNγ: MBS2601171, TNF-α: MBS7612133, and IL-4: MBS733925, My BioSource, San Diego, USA,). The nitic oxide (NO) and lysosome activity were detected as described by the methodology of Rajaraman et al. [32] and Sun et al. [33], respectively. The concentration of amyloid A was assessed with commercially available sandwich ELISA kits (Biosource, Camarillo, CA, USA). Glutamyl transferases (GGT) and lactate dehydrogenase (LDH) were colormetrically analyzed using kits obtained from Bio-diagnostic Company (Giza, Egypt). All laboratory analyses and biochemical assessments followed the ISO/IEC 17025 protocols (the last version in 2005). More details on the standards and yields (detection range, sensitivity, and inter- and intra-assays precision) of ELISA kits used in the experiment are presented in Table S1.

### 2.7. Economic Efficiency

Economic efficiency (EE, %) comprising both costs and net revenue was estimated. The dominant prices by USD of the experimental diets and rabbit’s meat in Egypt during the experimental period were as follows: price of 1 kg/live body weight on selling was 2.5 USD, 1 kg a feed cost was 0.33 USD, and 1 mL MEONE was 0.031 USD. All collected prices were calculated form the local market, then adjusted as the exchange rate from Egyptians Pounds to Dollars.

These data were used to estimate the following economic items as follows: Total feed costs = total FI per rabbit × price/kg. Net revenue = price of rabbit-total feed cost, and EE = net revenue/total feed cost.

### 2.8. Statistical Model and Analysis Procedure

The Levene and Shapiro–Wilk tests were conducted in order to check for normality and homogeneity of variance [34]. One-way anova of statistical analysis system (Proc Anova; SAS, 2012 version 8, Cary, NC, USA) was used for assessing growth performance, feed utilization, blood biochemical, oxidative DNA marker, and economic efficiency, Multiple comparisons among means were carried out by the Duncan’s Multiple Range Test. Results were expressed as means ± SE. statistical significance was accepted at *p*-value < 0.05. Figures were fitted by the Graph-Pad Prism software 9.0 (Graph Pad, USA).

## 3. Results

### 3.1. Meteorological Parameters

The overall means of ambient temperature (AT), relative humidity (RH), and temperature–humidity index (THI) during the whole experimental period were 30.91 ± 0.14 °C, 72.01 ± 0.64%, and 29.47 ± 0.11, respectively (Table 2). The THI obtained in the present study indicated that the growing rabbits suffer from severe heat stress.

### 3.2. Characterization of Marjoram Essential Oil Nanoemulsion Formation

**Figure 1:**
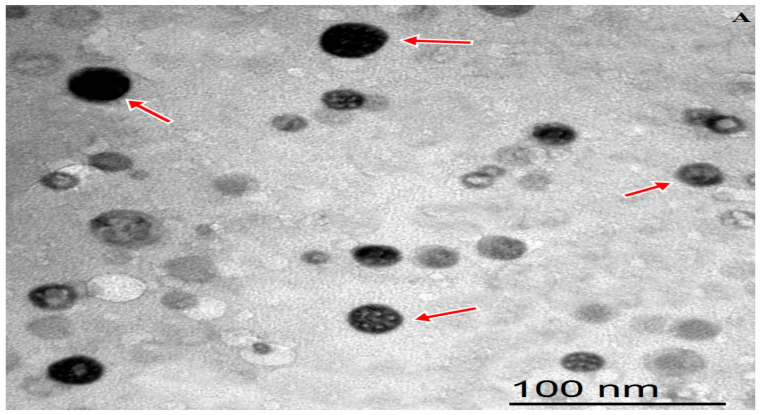

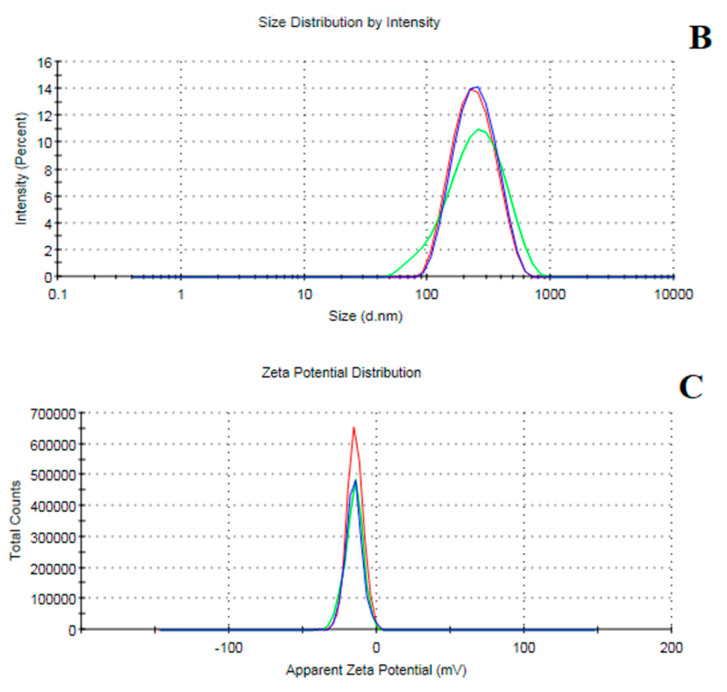
The morphology of MEONE by TEM (**A**), the particles size (**B**), and zeta potential distribution (**C**) of MEONE.

The TEM image for MEONE is presented in Figure 1A. The image shows spherical particles morphology of the tested nano-emulsion oils with little or no aggregation identified. The mean of particles size was 215 nm (Figure 1B). The values of PDI and Z-potential were 0.177 and −15.6 mV, respectively (Figure 1C).

### 3.3. Growth Performance, Feed Utilization, and Physiological Responses

**Figure 2:**
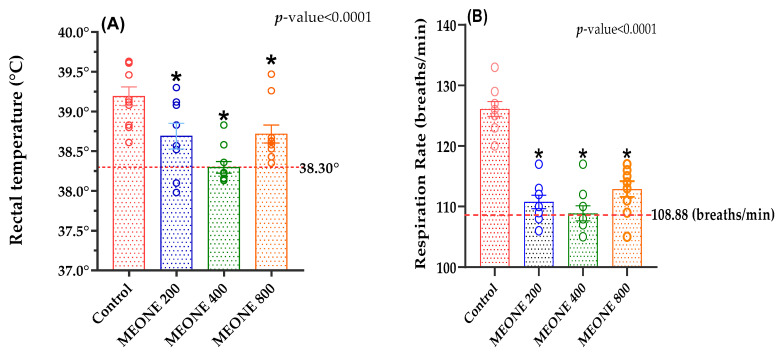
Overall mean of rectal temperature (**A**) and respiration rate (**B**) of heat-stressed rabbits fed dietary supplemented with 200 (MEONE200), 400 (MEONE400), and 800 (MEONE800) mg/kg of marjoram essential oil nanoemulsion (MEONE) compared to control. * *p* < 0.05: differ significantly with control.

Results shown in Table 3 revealed that the FBW was significantly increased in the MEONE200 and MEONE400 groups compared to the control group, whereas MEONE800 resulted in an intermediate value. Treatment with MEONE400 resulted in the highest significant PI and ADG. This improvement was observed at week 10 of age and afterwards, compared to other groups and control group (Table 3). However, FI was not affected by the treatment; FCR (lower FCR) was significantly improved in all treated groups compared with control group (Table 3). Treatment with MEONE400 significantly reduced RT and RR compared to the control group (Figure 2A,B).

### 3.4. Carcass Traits

Results in Table 4 show the carcass traits of heat-stressed rabbits fed different levels of MEONE compared with control rabbits. Only the dressing percentage and liverrelative weight were significantly affected by the dietary treatment (*p* = 0.0350 and 0.0296, respectively). The dressing percentage was significantly higher in the MEONE200 and MEONE400 groups than the control group (*p* < 0.05). Meanwhile, the relative weight of liver and total edible giblets were significantly higher in MEONE400 groups than their counterparts in the control group (*p* < 0.05).

### 3.5. Redox Status

The results in Table 5 show significant effects of dietary inclusion of MEONE on both of oxidative and anti-oxidative stress variables of heat-stressed growing rabbits, the lowest values of MDA and MYO were recorded in the MEONE800 group; however, the lowest values of PC were observed in the MEONE400 group compared to the HS-group (*p* < 0.05). With respected to the anti-oxidative stress markers, the elevated values of SOD and GSH were observed in the MEONE400 and MEONE800 groups, respectively. Meanwhile, the values of TAC maximized in the MEONE400 group. Despite the MEONE level, treatment with MEONEDNA significantly decreased oxidative marker (Ohdg) compared to the control group (Table 5).

### 3.6. Immunity and Inflammatory Responses and Liver Function

Dietary supplementation with different levels of MEONE significantly enhanced the IgG and IgM levels (*p* < 0.001), recording the greatest values in the MEONE800 group (*p* < 0.05; Table 6). With regard to inflammatory cytokines, the addition of MEONE declined the concentrations of IL-4 (*p* = 0.0003), IFN Y (*p* = 0.0004), TNF-α (*p* < 0.0001), and amyloid A (*p* < 0.0001) in the serum, but significantly increased the activity of NO (*p* = 0.0001), and LZM (*p* < 0.0001; Table 6). Heat stressed rabbits fed diets supplemented with MEONE (200, 400, or 800 mg MEONE/kg) had lower GGT and LDH compared to non-supplemented rabbits (Table 6).

### 3.7. Economic Efficiency

Results of economic efficiency are shown in Table 7. There were pronounce significant effects of dietary supplementation with MEONE on economic efficiency; the highest values of NE, economic efficiency, and relative economic efficiency were achieved by rabbits given a diet enriched with 400 mg MEONE, followed by 200 mg MEONE and 800 mg MEONE, while the control group achieved the lowest corresponding values.

## 4. Discussion

In tropical/subtropical regions, the harmful effects of HS on livestock are not easy to mitigate, specifically when the high ambient temperature is combined with high humidity. Therefore, breeders may need to apply more than one strategy (controlling housing system, nutritional manipulation, selection of heat tolerance animals) to protect rabbits against negative consequences of HS. Among HS alleviating strategies, nutritional interventions can present practical and effective solutions either applied alone or in combination with other alleviating strategies [7]. In this sense, several studies recommended the enrichment of diets of heat-stressed growing rabbits with phytogenic supplementation, including essential oils. Essential oils, such as marjoram, have many therapeutic activities, including antioxidants, anti-inflammatory, antimicrobial, antiviral, spasmolytic, carminative, and hepatoprotective effects [35], making them an ideal feed supplement for early weaned rabbits. Despite the beneficial biological effects of essential oils on animal health, these photogenic substances have some limitations related to its bioavailability and other industrial limitations [36,37]. Most essential oils are lipophilic molecules, and therefore their absorbance along the gastrointestinal tract can be improved if they are offered as emulsions. In this study, we used oil-in-water single-layer nanoemulsion procedure to fabricate MEOE in nanoform. The results of the physichochemical properties (size ≈ 200 nm and negative zeta potential, Z-potential ≈ −11 mV) confirmed the relevance of the resultant nanoemulsion for better intestinal absorbance and cellular uptake, increasing the bioavailability of the active components of MEOE. In this sense, small size, low-magnitude negative charge, and moderate hydrophilicity help nanoparticles pass through the small intestinal mucus layer more easily [38]. It has been found that the anionic nanoparticles induce tight junction relaxation, increasing intestinal permeability. This permeation-enhancing effect is a function of nanoparticle size and charge, with smaller (≤200 nm) and more negative particles conferring enhanced permeability [39].

In the present study, the THI value indicated that the growing rabbits are exposed to severe heat stress (28.9 to <30.0; [7]. Nevertheless, MEONE-supplemented rabbits possessed better heat tolerance capacity, growth performance, and health status (lower oxidative and inflammatory stress indicators). The administration of MEONE decreased the RT of growing rabbits exposed to serve heat stress, what in general agreement with several previous studies observed significant effects of natural antioxidants in decreasing the rectal temperature in rabbits due to its contents from bioactive components, such as flavonoids and flavones [39,40]. Moreover, in hot environments, rabbits begin to increase their respiratory activity in order to maintain their heat balance and, thus, losing more body heat throughout t evaporation from the respiratory tract, which explains the higher respiratory rate observed in control group compared to MEONE treated group, which also suggest the action of MEONE in heat regulation. These findings can also explain the improvement in growth performance of MEONE-supplemented rabbits, as these rabbits did not expenditure energy in heat regulation process, respiratory evaporation; instead, most of available energy is directed to the important biological functions, such as growth. This can be confirmed by the findings of our study as the MEONE-supplemented rabbits had better FBW, ADG, PI, and FCR compared to the control rabbits, recording the highest significant values with 400 mg MEONE/kg diet. The current results coincide with the results of Abdelhadi et al. [41], who showed significant effects of adding essential oils and nanoemulsified essential oils (garlic, pomegranate, and tea tree essential oils) to rabbit diets on growth performance and feed utilization. The improved growth performance in this study could be linked to the enhanced feed utilization and the nutrients digestibility. Essential oils perform a vital role in boosting the digestion through stimulating the secretion of digestive enzyme and fluids and improving guts eubiosis [42]. In this context, the relative weight of liver was significantly increased by MEONE400 supplementation. These results may signalize that the addition of MEONE in rabbit diets leads to improve the health status of liver, which performs a decisive role in synthesis of blood protein and other enzymes linked to heat tolerance [6,43,44].

It is well known that the high AT commonly upregulating the synthesis of free radicals and cytokines, causing oxidative and/or inflammatory stress [45]. Therefore, in this study, we have focused on studying the changes in these two pathways in heat-stressed and MEONE-supplemented rabbits. Giving in mind that essential oils can alleviate the negative effects of HS through controlling these two major cells damaging pathways, oxidative and inflammatory pathways [6,14,41,46,47,48].

The current study indicated that the fortification of heat-stressed rabbit diets with MEONE at high doses (800 mg/kg diet) decreased oxidative stress by lowering MDA and MYO levels by 30.59 and 87.31%, respectively. However, the concentration of PC decreased by 46.41% in the MEONE400 treated group compared to the control group. In the same manner, the evidence of anti-oxidative stress improved by the addition of MEONE to rabbit diets; the levels of TAC and GSH maximized in the high-dose treated group, while the activity of SOD maximized in MEONE400 treated group. In accordance with the present results, several former studies indicated that the essential oils with its bioactive component, including flavonoids and flavones, could enhance the oxidative stability and effectually deferring the lipid oxidation or proteins (protein carbonyl) and other nutrients by inhibiting the diffusion of oxidation reactions [49,50]. Interestingly, it was observed that the protective effects of MEONE extended also at molecular level, as MEONE administration reduced the concentrations of serum 8-OHdG, a new biomarker for the oxidative damage of DNA. Thus, it can be concluded that MEONE confers animals an integrated oxidative defense system that helps them to easily tolerate with oxidative stress evoked by severe heat stress.

Under heat stress conditions, a significant impairment in immune function can be observed. This is mainly mediated by the hypothalamic-pituitary-adrenal axis [51], which evokes the glucocorticoid functions as an anti-immune response element [52]. The increase in glucocorticoid concentration impairs both cellular and humoral immune systems. Consequently, the heat stress causes major losses in rabbit production due to the deterioration in the immune function that makes rabbits susceptible to pathogens [7]. In this study, the additions of MEONE in the diets of early weaned growing rabbits improve cellular and humoral immune systems. Rabbits supplemented with 400 or 800 mg MEONE/kg diet had significantly improved levels of IgG and IgM (cellular immunity) compared to rabbits in other groups. Moreover, the supplementation of rabbit diets with MEONE resulted in significant improvements in humoral immunity, as indicated by the increased lysosomal activity and concentration of amyloid A. The improved lysozyme activity may contribute to the elimination of pathogens because of its enzymatic degenerative potential [48,53], and amyloid A has a significant immunological activity either by being chemotactic for mast cells and neutrophils or by stimulating the synthesis of cytokines [54]. These positive effects of MEOE on immune system can be attributed to its wide biological activities, including anti-allergic, anti-inflammatory, antiviral, and antimicrobial activities [55].

Regarding inflammatory pathways, it is interesting to note that MEONE had the ability to suppress the de novo of inflammatory cytokines and, thus, inflammation reactions. High AT leads to an increase in the apical contents of pro-inflammatory cytokines, such as IFNγ and IL-4, which, in turn, raise the permeability of intestine of the animal to pathogens [56]. According to the findings of this study, MEONE-treated rabbits had a significant decrease in the pro-inflammatory cytokines (IL-4, TNF-α, and IFNγ), indicating the potential anti-inflammatory activity of MONE. On the other hand, MONE treatments resulted in signification increases in NO concentrations. Under physiological concentrations, this small molecule can act as an anti-inflammatory agent, neurotransmitter, immune regulatory factor, and vasodilator [57]. Recently, it has been concluded that NO can show an indispensable role in defending animals during exposure to adverse pathogen attacks or environmental conditions throughout the enhancing of non-specific immunity [14,58]. Moreover, the vasodilation role of NO can partially explain the improved heat tolerance ability (lower RT and RR) of MEONE-supplemented rabbits. The re-partitioning of the blood toward peripheral blood vesicles (e.g., ear blood vesicles in rabbits) is an important physiological process for body heat loss. Interestingly, the present results indicated that the high level of NO in the blood serum of growing rabbits treated by MEONE is associated with increases in the antioxidant markers, such as GSH and TAC. This supports that the NO concentrations were at physiological levels, as NO is classified as a free radical as well.

Our results indicated the safety of the MEONE to growing rabbits as indicated by the liver function. High levels of gamma glutamyl transferase (GGT) in the blood may be a sign of liver impairment (disease or damage to the bile ducts). Former studies have been indicated that high ambient temperature elevates the levels of GGT may be due to disturbance of enzyme synthesis, damage in the liver, or changes in the permeability of hepatic cells membrane, which causes the enzymes to leak into the bloodstream [59]. In the current study, MEONE has demonstrated a healthy defensive property against heat stress-induced hepatotoxicity by reducing the mentioned enzymatic activity.

It is known that under subtropical conditions, HS has adverse impacts on the growth performance of rabbits, leading to major economic losses due to high rates of animals mortality, low feed efficiency, and costs related to health management [60]. In this study, the inclusion of MEONE in the diets of growing rabbits contributes to raising the economic values of the supplemented diets, making the fattening process more profitable.

## 5. Conclusions

To summarize, exposing growing rabbit to heat stress negatively affect growth; feed utilization; meat quality; health status, mainly via the elevation of oxidative stress and inflammatory responses; and the impairment of immune system functions. Under the conditions of our study, supplementation with 400 mg MEONE/kg diet improved growth and feed efficiency and the profitability. Moreover, serum antioxidant and anti-inflammatory capacities were improved, which are associated with adequate immune functions. These effects may be due to the improved bioavailability of MEOE after fabricating in a nano form. Further advanced studies are needed to explore the manner by which these nanoparticles are absorbed and circulating to emphasize the actual need for nanomulsion technology in phytogenic feed additives processing. Moreover, active components of MEOE and their roles at cellular/molecular levels as active thermoregulatory agents need more investigations.

## Figures and Tables

**Table 1:** The ingredients and chemical composition of the basal diet.

Items	Basal Diet
Ingredient	%
Berseem hay	30
Wheat bran	16
Barley grain	10
Maize	20
Soybean meal	20
Molasses	2
Limestone	1
NaCl	0.5
Premix *	0.5
Calculated composition, % **	
Metabolizable energy, MJ/kg	7.95
Crude protein	17.50
Calcium	0.88
Available phosphorus	0.20

* Each 1 kg of premix (minerals and vitamin mixture) comprises vit. K, 0.33 g; vit. B1, 0.33 g; vit. D3, 15,000 IU; vit. B2, 1.0 g; vit. pantothenic acid, 3.33 g; vit. B5, 8.33 g; vit. B12, 1.7 mg; B6, 0.33 g; biotin, 33 mg; folic acid, 0.83 g; choline chloride, 200 g; vit. A, 20,000 IU; vit. E, 8.33 g; ** Calculated according to NRC [29]. The diet is free of antibiotics.

**Table 2:** The mean values of temperature–humidity index, relative humidity, and ambient temperature during the trial duration.

Parameters	July	August	Overall
AT (°C)	31.09 ± 0.15	30.73 ± 0.21	30.91 ± 0.14
RH (%)	72.10 ± 0.88	71.91 ± 1.07	72.01 ± 0.64
THI	29.65 ± 0.11	29.31 ± 0.16	29.47 ± 0.11

AT: ambient temperature; RH: relative humidity; THI: temperature–humidity index.

**Table 3:** Growth performance and feed utilization of heat-stressed rabbits fed dietary supplemented with 200 (MEONE200), 400 (MEONE400), and 800 (MEONE800) mg/kg of marjoram essential oil nano-emulsion (MEONE) compared to control (mean ± SE).

Items	Control	MEONE200	MEONE400	MEONE800	*p*-Value
Body weight (g)					
At 6 wks of age	704.18 ± 24.07	703.75 ± 42.93	708.83 ± 30.53	711.25 ± 26.80	0.9842
At 10 wks of age	1401.03 ± 24.69 ^b^	1483.63 ± 31.76 ^a,b^	1527.50 ± 19.34 ^a^	1472.13 ± 33.49 ^a,b^	0.0490
At 14 wks of age	2007.50 ± 44.79 ^b^	2183.25 ± 41.69 ^a^	2210.86 ± 33.01 ^a^	2142.85 ± 56.93 ^a,b^	0.0339
Daily weight gain (g)					
6–10 wks of age	24.89 ± 0.79 ^b^	27.85 ± 0.97 ^a,b^	29.24 ± 0.92 ^a^	27.17 ± 1.09 ^a,b^	0.0522
10–14 wks of age	21.66 ± 0.87 ^b^	24.99 ± 0.98 ^a^	24.41 ± 0.86 ^a,b^	23.95 ± 1.13 ^a,b^	0.0345
Overall (6–14 wks of age)	23.27 ± 0.88 ^b^	26.42 ± 0.97 ^a,b^	26.82 ± 0.89 ^a^	25.56 ± 1.10 ^a,b^	0.0275
Feed intake (g/day)					
6–10 wks of age	71.54 ± 3.91	66.98 ± 3.19	68.01 ± 1.61	71.81 ± 2.08	0.5382
10–14 wks of age	112.09 ± 7.09	116.09 ± 10.25	104.94 ± 5.12	104.70 ± 3.83	0.6040
Overall (6–14 wks of age)	91.80 ± 2.07	91.54 ± 3.81	86.47 ± 3.19	88.26 ± 1.47	0.4873
Feed conversion ratio (g feed/g gain)					
6–10 wks of age	2.87 ± 0.24	2.41 ± 0.19	2.33 ± 0.07	2.64 ± 0.17	0.1012
10–14 wks of age	5.17 ± 0.15 ^a^	4.64 ± 0.23 ^b^	4.30 ± 0.16 ^b^	4.37 ± 0.12 ^b^	0.0154
Overall (6–14 wks of age)	3.94 ± 0.11 ^a^	3.46 ± 0.05 ^b^	3.22 ± 0.10 ^b^	3.45 ± 0.13 ^b^	0.0036
PI (%)	51.77 ± 5.32 ^b^	66.86 ± 5.88 ^a,b^	67.23 ± 2.55 ^a^	60.49 ± 5.49 ^a,b^	0.0382

^a,b^ Means in the same row with different superscript letter following them are significantly different (*p* < 0.05).

**Table 4:** Carcass traits of heat-stressed rabbits fed dietary supplemented with 200 (MEONE200), 400 (MEONE400), and 800 (MEONE800) mg/kg of marjoram essential oil nano-emulsion (MEONE) compared to control. Results were presented as mean ± SE.

Items	Control	MEONE200	MEONE400	MEONE800	*p*-Value
Pre-slaughter weight (g)	2021.67 ± 60.09	2153.33 ± 41.06	2181.11 ± 20.20	2093.67 ± 40.53	0.1148
Carcass Traits (as % of pre-slaughter BW)					
Dressing	57.72 ± 0.94 ^b^	61.20 ± 0.97 ^a^	61.62 ± 0.86 ^a^	59.08 ± 0.80 ^a,b^	0.0350
Head	4.95 ± 0.09	5.14 ± 0.36	5.36 ± 0.12	4.85 ± 0.08	0.3247
Liver	3.20 ± 0.11 ^b,c^	3.57 ± 0.25 ^a,b^	3.73 ± 0.12 ^a^	3.06 ± 0.10 ^c^	0.0296
Heart	0.24 ± 0.02	0.30 ± 0.03	0.33 ± 0.04	0.31 ± 0.04	0.2837
Kidney	0.61 ± 0.02	0.56 ± 0.03	0.58 ± 0.05	0.57 ± 0.05	0.8871
Edible Giblets	3.99 ± 0.11 ^b^	4.44 ± 0.20 ^a,b^	4.64 ± 0.15 ^a^	3.91 ± 0.12 ^b^	0.0090
Spleen	0.09 ± 0.01	0.08 ± 0.01	0.10 ± 0.02	0.08 ± 0.01	0.6308
Lung	0.61 ± 0.02	0.59 ± 0.06	0.59 ± 0.05	0.64 ± 0.02	0.7912
Cecum Length (Cm)	11.55 ± 0.26	11.76 ± 0.49	11.63 ± 0.27	11.59 ± 0.71	0.2345

^a,b,c^ Means in the same row with different superscript letter following them are significantly different (*p* < 0.05).

**Table 5:** Redox status of heat-stressed rabbits fed dietary supplemented with 200 (MEONE200), 400 (MEONE400), and 800 (MEONE800) mg/kg of marjoram essential oil nano-emulsion (MEONE) compared to control. Results were presented as mean ± SE.

Items	Control	MEONE200	MEONE400	MEONE800	*p*-Value
Oxidative biomarkers					
MDA (nmol/mL)	0.487 ± 0.015 ^a^	0.347 ± 0.022 ^b^	0.343 ± 0.019 ^b^	0.320 ± 0.007 ^b^	0.0164
MYO (ng/mg protein)	6.800 ± 0.450 ^a^	5.397 ± 0.247 ^a^	2.423 ± 0.043 ^b^	2.117 ± 0.035 ^b^	<0.0001
PC (nmol/mL)	2.657 ± 0.032 ^a^	2.040 ± 0.064 ^b^	1.332 ± 0.030 ^c^	1.421 ± 0.089 ^c^	<0.0001
Anti-oxidative enzymes					
TAC) U/mL)	1.264 ± 0.029 ^b^	1.314 ± 0.021 ^a,b^	1.532 ± 0.067 ^a^	1.195 ± 0.007 ^b^	0.0001
SOD (ng/mL)	1.960 ± 0.017 ^b^	2.348 ± 0.043 ^b^	3.710 ± 0.040 ^a^	3.948 ± 0.114 ^a^	0.0001
GSH (ng/mL)	0.236 ± 0.011 ^c^	0.211 ± 0.012 ^c^	0.668 ± 0.004 ^a^	0.550 ± 0.014 ^a^	0.0001
Ohdg (pg/mL)	2.812 ± 0.258 ^a^	2.306 ± 0.095 ^b^	0.831 ± 0.026 ^c^	0.886 ± 0.034 ^c^	0.0001

^a,b,c^ Means in the same row with different superscript letter following them are significantly different (*p* < 0.05). MDA: malondialdehyde; MYO: myeloperoxidase; PC: protein carbonyl; TAC: total antioxidant capacity; SOD: superoxide dismutase; GSH: glutathione; Ohdg: DNA oxidative marker (8-hydroxy-2′–deoxyguanosine).

**Table 6:** Immunity, inflammatory cytokines, and liver enzymes of heat-stressed rabbits fed dietary supplemented with 200 (MEONE200), 400 (MEONE400), and 800 (MEONE800) mg/kg of marjoram essential oil nano-emulsion (MEONE) compared to control. Results were presented as mean ± SE.

Items	Control	MEONE200	MEONE400	MEONE800	*p*-Value
Immunity					
IgG (ng/mL)	38.431 ± 3.756 ^c^	49.670 ± 4.096 ^b^	59.125 ± 1.202 ^b^	66.862 ± 1.732 ^a^	0.0005
IgM (ng/mL)	71.662 ± 1.044 ^b^	74.670 ± 1.814 ^b^	83.209 ± 3.606 ^a^	88.526 ± 1.848 ^a^	0.0003
Inflammatory cytokines					
NO (Umol/L)	41.012 ± 0.542 ^b^	63.027 ± 2.887 ^a^	69.610 ± 2.028 ^a^	61.630 ± 2.333 ^a^	0.0001
TNF-α (pg/mL)	77.700 ± 5.331 ^a^	45.230 ± 1.760 ^b^	49.000 ± 3.215 ^b^	42.310 ± 2.111 ^b^	<0.0001
IL-4 (pg/mL)	102.312 ± 3.844 ^a^	87.671 ± 2.111 ^b^	74.322 ± 1.202 ^c^	73.110 ± 1.411 ^c^	0.0003
IFN-γ (pg/mL)	76.224 ± 2.331 ^a^	49.667 ± 1.764 ^b^	46.121 ± 2.082 ^b^	51.236 ± 2.186 ^b^	0.0004
LZM (pg/mL)	1.632 ± 0.041 ^c^	2.025 ± 0.064 ^b^	3.331 ± 0.081 ^a^	3.263 ± 0.026 ^a^	<0.0001
Amyloid A (ng/mL)	4.714 ± 0.204 ^a^	4.097 ± 0.124 ^b^	2.167 ± 0.051 ^c^	1.330 ± 0.066 ^d^	<0.0001
Liver enzymes					
GGT (mg/dL)	55.225 ± 1.451 ^a^	45.217 ± 0.337 ^b^	26.528 ± 2.604 ^c^	29.562 ± 0.883 ^c^	<0.0001
LDH (mg/dL)	138.530 ± 5.547 ^a^	102.211 ± 3.282 ^b^	80.335 ± 1.452 ^c^	84.453 ± 4.255 ^c^	<0.0001

^a,b,c^ Means in the same row with different superscript letter following them are significantly different (*p* < 0.05). IgG: immunoglobulin G; IgM: immunoglobulin M; NO: nitric oxide; TNF-α: tumor necrosis factors; IL-4: interleukin-4; IFN Y: interferon Y; LZM: Lysozyme; GGT: glutamyl transferase; LDH: lactate dehydrogenase.

**Table 7:** Economic efficiency of heat-stressed rabbits fed dietary supplemented with 200 (MEONE200), 400 (MEONE400), and 800 (MEONE800) mg/kg of marjoram essential oil nano-emulsion (MEONE) compared to control. Results were presented as mean ± SE.

Items	Control	MEONE200	MEONE400	MEONE800
Live body weight (kg)	2.007 ± 0.044 ^b^	2.183 ± 0.041 ^a^	2.210 ± 0.033 ^a^	2.142 ± 0.057 ^a,b^
BWG/Rabbit (kg)	1.312 ± 0.049	1.478 ± 0.054	1.500 ± 0.050	1.428 ± 0.061
Total revenue/rabbit (USD)	3.413 ± 0.123	3.844 ± 0.136	3.900 ± 0.125	3.715 ± 0.154
Feed intake/rabbit (kg)	5.141 ± 0.116	5.126 ± 0.213	4.842 ± 0.178	4.942 ± 0.082
Feed cost/rabbit (USD)	1.696 ± 0.038	1.677 ± 0.061	1.598 ± 0.059	1.630 ± 0.027
Oil cost/rabbit (USD)	0.000 ± 0.000	0.034 ± 0.001	0.063 ± 0.001	0.130 ± 0.001
Total costs/rabbit (USD)	1.692 ± 0.039	1.711 ± 0.061	1.662 ± 0.059	1.761 ± 0.027
NE/rabbit (USD)	1.808 ± 0.103 ^b^	2.133 ± 0.088 ^a^	2.238 ± 0.099 ^a^	1.953 ± 0.144 ^a^
Economic efficiency (USD)	1.067 ± 0.054 ^b^	1.247 ± 0.039 ^a^	1.350 ± 0.069 ^a^	1.109 ± 0.082 ^a^
REE	100	116.869	126.523	103.936

^a,b^ Means in the same row with different superscript letter following them are significantly different (*p* < 0.05). BWG: body weight gain; REE: relative economic efficiency = net revenue/total feed cost on selling was 2.5 USD, 1 Kg feed costs was 0.33 USD, and 1 mL MEONE was 0.031 USD.

## Data Availability

The data of this research are available upon request.

## Supplementary Materials

The following supporting information can be downloaded at: https://www.mdpi.com/article/10.3390/ani13111772/s1, Table S1: The standards and yields of ELISA kits used in the experiment.
