# Supplementary material for: Struggling Thermal Stress Impacts on Growth Performance and Health Status of Newly Weaned Rabbits Using Nanoemulsion of Origanum majorana Considering the Economic Efficiency of Supplementation
Source: Animals (Basel). 2023 May 26;13(11):1772. doi: 10.3390/ani13111772 (PMC10252083; doi:10.3390/ani13111772)
Supplement: Supplementary file 1 [file animals-13-01772-s001.zip › animals-2292270-supplementary.pdf]

Table S1. the standards and yields of ELISA kits used in the experiment.

ELISA Kit name	Detection Range	Sensitivity	Intra-assay Precision	Inter-assay Precision
MBS8807700	15.63-1000 pg/mL	6.5 pg/mL	CV%<8%	CV%<10%
MBS8807589	0.16-10 U/mL	0.066 U/mL	CV%<8%	CV%<10%
MBS9718983				
MBS8806802	31.25-2000 ng/mL	9.15 ng/mL	CV%<8%	CV%<10%
MBS724170	2.5-50ng/mL	0.1ng/mL	--	CV%<10%
MBS2601171	1000 pg/mL-15.6 pg/mL	Up to 5 pg/mL	<= 8%	<= 12%
MBS7612133	7.813-500pg/ml	4.688pg/ml	CV%<8%	CV%<10%
MBS733925	5-500pg/ml	1.0 pg/mL		
